# Leiomyosarcoma of the spermatic cord: a rare paratesticular neoplasm case report

**DOI:** 10.1186/s12957-022-02539-9

**Published:** 2022-03-25

**Authors:** Farah Ahmed, Asadullah Aslam, Yousaf Tanveer, Syed Jaffry

**Affiliations:** 1 Department of Urology, Letterkenny University Hospital, Letterkenny, Ireland; 2 Cavan General Hospital, Cavan, Ireland; 3 Department of Urology, Galway University Hospital, Galway, Ireland

**Keywords:** Spermatic cord leiomyosarcoma, Paratesticular neoplasm, Scrotal mass, Case report

## Abstract

**Background:**

Primary soft tissue sarcomas contribute to only 2% of all malignancies arising from the male genitourinary tract. Leiomyosarcoma (LMS) is a malignant soft tissue neoplasm which originates from the mesenchyme and has a characteristic smooth muscle differentiation. Usually, it presents as a painless, firm, slow-growing unilateral scrotal mass. Investigations include imaging, tumor markers, and histopathology.

**Case presentation:**

A 65-year-old gentleman known diabetic and beta-thalassemic trait was referred to the Urology OPD at Letterkenny University Hospital. His presenting complaint was a left groin lump that appeared 1 year ago and was growing larger in size gradually. According to the patient, his lump was slightly painful (localized) initially that later became painless. He did not report any testicular trauma/infection or UTI. There was no significant history of malignancies running through his family. Clinical examination revealed a soft and lax abdomen, normal testes. There was a non-tender 2cm x 2cm well-circumscribed, mobile, firm to cystic irreducible left inguinoscrotal mass and appeared to be attached to the spermatic cord. Cough impulse was indiscernible. Ultrasound left groin showed 1.8 cm transverse x 1.4 cm AP x 1.9 cm sagittal) well-circumscribed ovoid nodular subcutaneous lesion present in the upper left inguinal area just lateral to the left pubic tubercle that appeared solid with heterogeneous internal echotexture and no internal calcification. Some internal vascularity is demonstrated with color Doppler assessment.

**Conclusion:**

Because of its rareness, LMS represents a management conundrum. There is no standard protocol for treatment. We present a case and discuss the available evidence from the literature to date to help identify LMS of the spermatic cord that is highly unusual.

## Background

Masses in the scrotal sac in adults can be either testicular or paratesticular. Paratesticular tumors are uncommon and account for < 5% of scrotal masses. The paratesticular area can give rise to a vast range of neoplasms with a wide behavioral spectrum due to its complex anatomy which houses a number of structures, including the epididymis, tunica vaginalis, and spermatic cord [1].

Scrotal leiomyosarcomas (LMS) are rare. Approximately 30% are malignant and of those almost 90% are sarcomas [2]. Primary soft tissue sarcomas represent only 2% of all malignancies arising from the male genitourinary tract which makes them the rarest malignancy in that location [3]. Liposarcomas (LPS) are the most frequent, followed by leiomyosarcoma (LMS), rhabdomyosarcoma (RMS), fibrosarcoma (FS), and undifferentiated pleomorphic sarcoma (UPS) [1]. 75% of male genitourinary sarcomas originate in the spermatic cord [4].

The pre-operative diagnosis of LMS is difficult. Since most arise from the distal spermatic cord, distinguishing them clinically from a scrotal mass is challenging. LMS can be mistaken as epididymal cyst, cord lipoma, incarcerated hernia, inguinal hernia, and epididymo-orchitis. The clinical presentation is usually vague and non-typical. It can range from painless to mildly painful paratesticular intrascrotal mass with or without a feeling of heaviness [5]. The peak incidence is commonly in the 5th or 6th decade of life [6].

## Case presentation

A 65-year-old man with type-2 diabetes and a beta-thalassaemic trait was referred to urology presenting with a left groin lump. This had appeared about 1 year ago and gradually increased in size. Initially, the lump was slightly painful but later became painless.

There was no history of any testicular trauma, sexually transmitted diseases, UTI, undescended testis, or groin/scrotal surgery of any kind. There was no family history of malignancies.

Clinical examination revealed a soft abdomen and normal testes. There was a non-tender, 2x2cm well-circumscribed, mobile, firm left inguinoscrotal mass which appeared to be attached to the spermatic cord.

**Fig. 1:**
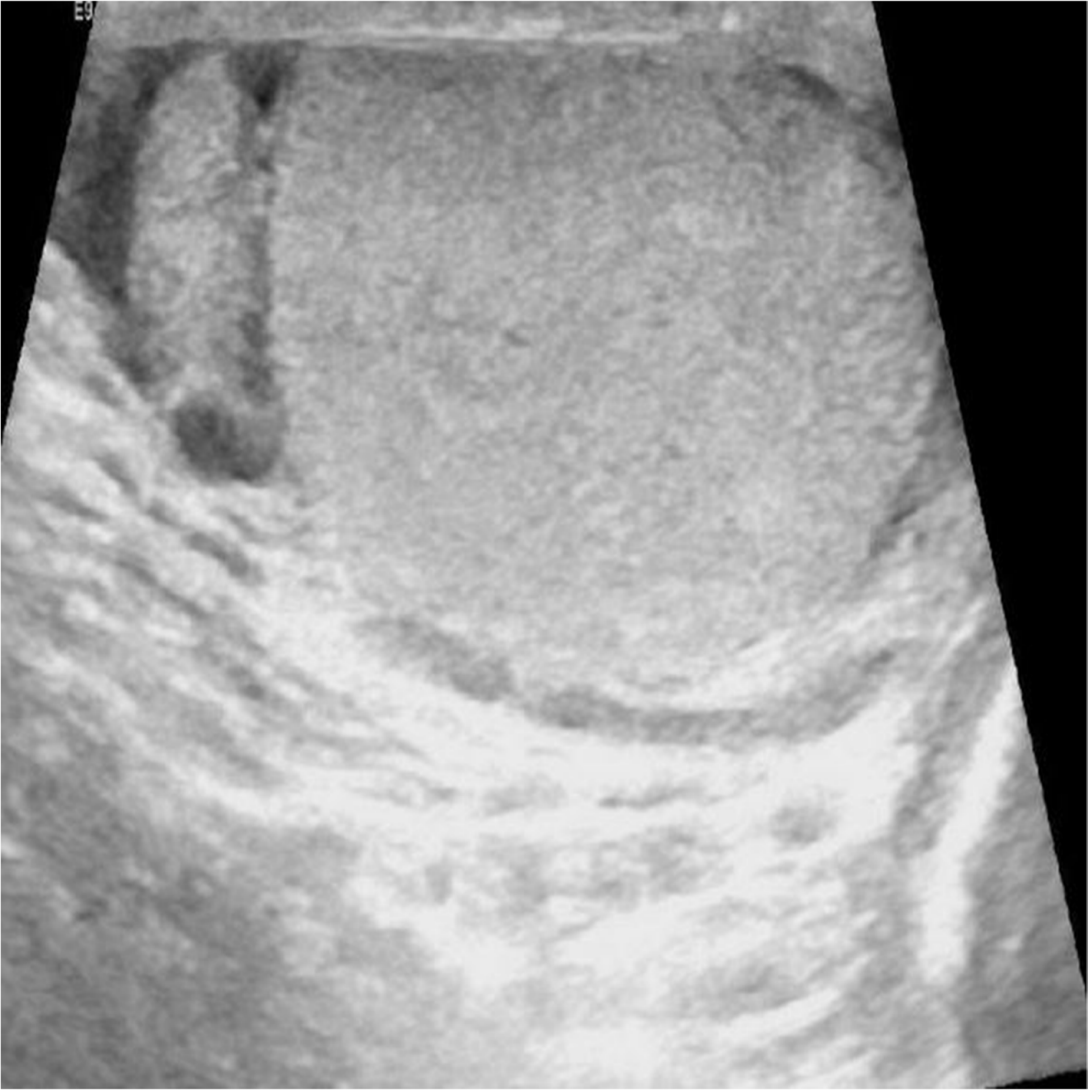
USS left groin demonstrating a well-circumscribed ovoid, solid, and vascular lesion, with heterogeneous internal echotexture

Ultrasound of the left groin (Fig. 1) showed a 1.8x1.4x1.9cm well-circumscribed ovoid nodular lesion in the upper left inguinal area just lateral to the left pubic tubercle. It appeared solid with a heterogeneous internal echotexture. There were no internal calcifications and internal vascularity was demonstrated with color Doppler assessment.

**Fig. 2:**
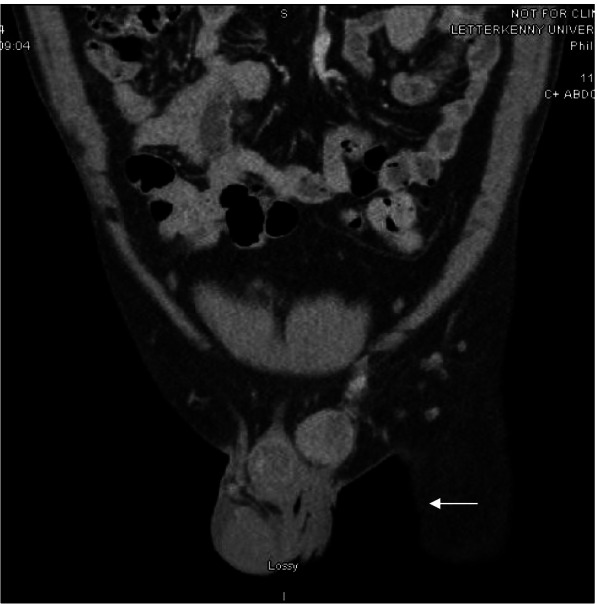
CT showing a mass in the left spermatic cord (arrow)

Three-phasic contrast CT (Fig. 2) showed the mass as 30 x 25 x 23mm, marginally larger than on ultrasound. The mass appeared to be continuous with the spermatic cord. No lymph node involvement or distant metastasis was demonstrated.

Laboratory parameters were all within normal limits. Testicular tumor markers such as beta-HCG, alpha-fetoprotein (AFP), and lactate dehydrogenase (LDH) were in the normal range.

**Fig. 3:**
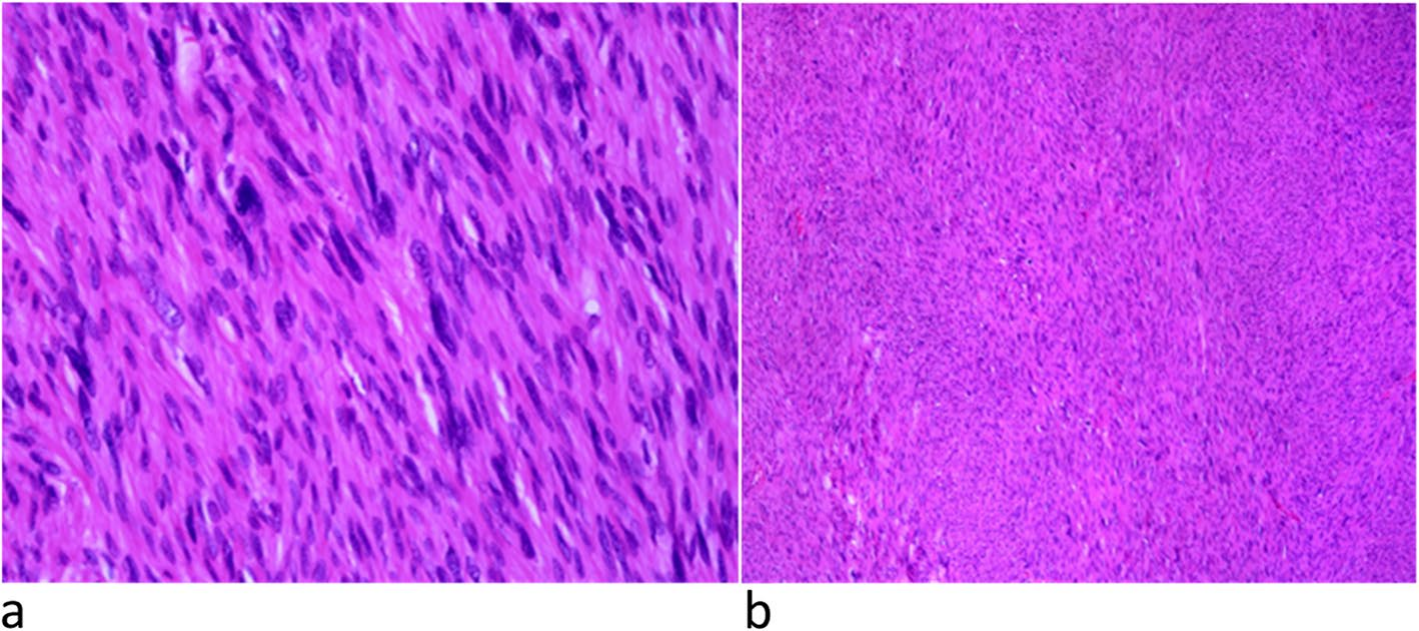
**a**, **b** Microscopy showing **a** nuclear atypia and **b** increased mitotic activity

The patient underwent a biopsy excision of the lesion. Histopathology showed spindle-shaped, elongated, moderately atypical cells in fascicular arrangement (Fig. 3a) with oval to elongated nuclei showing mild to moderate mitotic activity (Fig. 3b). On immunohistochemical staining, cells were positive for smooth muscle actin (SMA), muscle-specific actin (MSA), desmin, and negative for pancreatokeratin, S-100, and SOX10. There was no necrosis. These findings were strongly suggestive of LMS.

The French Federation of Cancer Centres Grading System for sarcomas was employed to further classify the specimen [5]. Each of its domains is mentioned below, along with their respective scores:

- Tumor differentiation: 2
- Mitotic count: 2
- Tumor necrosis: 0

With an overall score of 4, our specimen was suggestive of a Grade 2 LMS of the spermatic cord.

Consequently, the patient underwent left orchiectomy with excision of the spermatic cord up to the deep inguinal ring via an inguinal open approach.

The postoperative course was uneventful. The final histopathology of the orchiectomy specimen showed that the LMS had earlier been resected completely. Follow-up surveillance with the CT chest, abdomen, and pelvis did not reveal any recurrence, lymph nodes, or distant metastasis. Twelve months of follow-up did not reveal any recurrence. The patient was satisfied with the treatment given to him. Written informed consent was obtained from the patient for publication of this case report.

## Discussion

The first paratesticular sarcoma was reported in 1996 [7]. LMS is the second most common malignant soft tissue sarcoma with 19–32% of cases. It has a mesenchymal cell origin and smooth muscle differentiation. There is no evidence for a genetic abnormality in spermatic cord LMS [8]. The disease-specific survival rate is between 77% [8]. LMS of the spermatic cord is very rare with 113 cases reported to date worldwide [9].

LMS exhibits a wide-ranging clinical presentation. Clinically, any painless slow-growing lump should be considered suspicious for LMS amongst other differential diagnoses [9]. It is important to understand the process of dissemination. LMS can spread in three ways, namely loco-regionally, hematogenous, and via the lymphatic route. Local spread is the most common. Hematogenous dissemination generally is to the liver and lungs, while lymphatic spread involves the external iliac, hypogastric, para-aortic, and common iliac nodes [8].

As for any unclear suspicious testicular or para-testicular lesion, investigations for LMS should include initial ultrasonography, CT, testicular tumor markers, histopathology, and immunohistochemical staining.

Ultrasonography has excellent sensitivity and specificity to assess any lesion in the scrotum. It is easy accessibility, and safe application makes it the initial exam of choice [10]. Ultrasound can differentiate between testicular and para-testicular masses with a sensitivity of 95% [11]. CT can be helpful in differentiating a primary para-testicular lesion from a retroperitoneal process extending into the scrotum [5]. It has to be borne in mind that imaging alone cannot determine a tumor diagnosis and has the potential to lead to misdiagnosis [12].

The definite diagnosis relies on histopathology and immunohistochemistry [5]. The grading of para-testicular sarcoma depends on the number of mitosis per 10 high-power fields (HPF), the percentage of necrosis, and pleomorphism of nuclei [5]. For LMS, immunohistochemistry will confirm smooth muscle differentiation and stain positive with SMA, MSA, and desmin [12].

Because of its rarity, there is no consensus on the treatment for spermatic cord LMS [6]. Orchidectomy with spermatic cord excision up to the deep inguinal ring is recommended for resectable LMS [9]. Achieving negative histological margins is challenging due to anatomical constraints in the para-testicular region [5]. The exact extent to which local tissue should be resected is not defined to date [6]. Loco-regional recurrences are estimated to occur in 30–50%, often through contiguous extension and tissue infiltration [13]. There is no consensus on the role of lymph node dissection to prevent loco-regional spread despite a 29% potential nodal failure rate [14]. There is no evidence of the improvement of survival by adjunctive nodal dissection [12].

There is no guideline on radiotherapy for LMS of the spermatic cord. Some authors advocate the use of adjuvant radiotherapy after orchidectomy to reduce local disease recurrence [14].

Chemotherapy has no clearly defined role in the LMS of the spermatic cord. It is often employed in metastatic disease [6]. A long follow-up is recommended as the disease is known to relapse even after 15 years [15].

In conclusion, LMS must be considered in the differential diagnosis of a slow-growing, unilateral, para-testicular mass in middle-aged men.

Radical orchidectomy and high spermatic cord excision along with resection of surrounding soft tissues are required to achieve a negative margin. Lymph node dissection is only recommended in cases of enlarged lymph nodes. Chemotherapy and radiotherapy have a limited and as yet poorly defined role in specific cases only. And finally, a long follow-up is mandatory as late recurrences are possible.

## Data Availability

NA
